# First Evaluations and Cryopreservation of Semen Samples from Sunda Clouded Leopards (*Neofelis diardi*)

**DOI:** 10.3390/ani10061072

**Published:** 2020-06-22

**Authors:** Zainal Zahari Zainuddin, Mohamed Reza Mohamed Tarmizi, Keng Chee Yap, Comizzoli Pierre, Symphorosa Sipangkui

**Affiliations:** 1 Borneo Rhino Alliance, c/o Faculty of Science and Natural Resources, Universiti Malaysia Sabah, Kota Kinabalu 88400, Sabah, Malaysia; reza2727@gmail.com (M.R.M.T.); kcyap.vet.upm@gmail.com (K.C.Y.); 2 Smithsonian Conservation Biology Institute National Zoological Park, P.O. Box 37012, MRC 5502, Washington, DC 20013-7012, USA; comizzolip@si.edu; 3 Sabah Wildlife Department, Tingkat 4, Blok B, Wisma MUIS, Kota Kinabalu 88100, Sabah, Malaysia; danguard78@gmail.com

**Keywords:** Sunda clouded leopards, semen evaluation, teratospermia, ultrasonography

## Abstract

**Simple Summary:**

The Sunda clouded leopard is a medium-sized cat from Borneo and Sumatra. The captive population has less than 20 individuals on both islands. Conservation breeding of this species has been unsuccessful due to the small numbers of individuals and the difficulty of pairing them. The present study aimed at evaluating for the first time the reproductive organs, semen characteristics, and sperm freezing for two wild-caught males (8 and 11 year old) living singly. The morphometric and ultrasound examination showed some degree of abnormalities in both leopards. Semen evaluation obtained by electro-ejaculation revealed a high proportion of abnormal sperm cells (similar to mainland clouded leopards). While only the younger male produced samples that could be frozen, post-thaw evaluations showed that the few surviving spermatozoa could potentially be used for in vitro fertilization or sperm injection. Studies on more individuals are needed to validate those encouraging but preliminary results.

**Abstract:**

A better understanding of semen characteristics and resilience to freezing temperatures is necessary before developing assisted reproductive techniques and systematic biobanking for the Sunda clouded leopard. The objective of this study was to evaluate for the first time the semen and sperm quality (in fresh and frozen samples) of two captive Sunda clouded leopards in Malaysia. A total of 17 examinations of the reproductive tract (using ultrasonography) and electro-ejaculations were performed on the two leopards over a 2-year period. Samples obtained from Leopard 1 (8 years old) varied in terms of volume (402 ± 92 µL), pH (7.9 ± 0.9), sperm motility (54.5 ± 24.2%), sperm concentration (122.4 ± 84.7 × 10^6^ sperm/mL), normal morphology (23.9 ± 12.3%), and viability (55.2 ± 18.2%). Midpiece defects represented the most common structural abnormality followed by abnormal tail and head defects. Samples from Leopard 2 (11 year old with abnormal testicular tissue) were of lesser quality. Two frozen semen samples from Leopard 1 were thawed and examined for acrosome integrity. Post-thawed samples contained <10% of motile spermatozoa but almost 50% of abnormal acrosomes. The present results emphasized the high incidence of structurally-abnormal spermatozoa, similar to the mainland clouded leopard. Post-thaw evaluations showed that the few surviving spermatozoa could potentially be used for in vitro fertilization or sperm injection. However, more individuals must be studied to validate those first findings that are exciting but still preliminary.

## 1. Introduction

The genus *Neofelis* comprises one species of clouded leopard found in continental South-east Asia (*Neofelis nebulosa*) and one species living on the islands of Borneo and Sumatra (*N. diardi*)—the Sunda clouded leopard. The island species is more endangered than the mainland one, with an estimated 750 individuals in Sabah, Borneo, and is classified as endangered, under the International Union for Conservation of Nature Red List [1,2]. It also is the least studied species of large cats in terms of ecology, behavior, and reproduction. In general, clouded leopards reach sexual maturity between the ages of 20 and 30 months. The breeding season observed in Khao Kheow Open Zoo, Thailand is between November to March, with gestation period ranging from 85 to 121 days. Lifespan in captivity ranged from 13 to 15 years [3,4,5].

The population under human care has fewer than 20 individuals with no breeding success (Dr. Bongot Huaso Mulia, Taman Safari Indonesia, personal communication, 23 March 2020). Captive breeding of clouded leopards is challenging due to male–female aggression, which sometimes leads to the death of individuals [6]. The development of assisted reproductive techniques is therefore essential to propagate the population and ensure a good genetic diversity [7] However, there is a need to better understand reproductive biology, including semen characteristics, before developing reproductive biotechnologies [7]. Semen cryopreservation for systematic biobanking is also tightly associated with these efforts and must be explored in parallel [8]. Previous studies in mainland clouded leopards have revealed a high incidence of teratospermia with ≥60% of structurally-abnormal spermatozoa and a high incidence of pleiomorphism with a lot of coiled-tailed defects [6,9]. These issues are likely due to low gene diversity in a small captive population of *N. nebulosa* [9]. The most recent reports in semen cryopreservation in mainland clouded leopards have demonstrated that post-thawing survival is variable and low as an average [7].

The objective of this study was to evaluate semen and sperm characteristics of two captive Sunda clouded leopards living singly. In addition, we assessed the quality of frozen-thawed semen from one individual.

## 2. Materials and Methods

### 2.1. Animals and Husbandry

The study was conducted on two wild-caught, adult male Sunda clouded leopards, with institutional approval from Sabah Wildlife Department (via letter with reference number JHL (HQ)400-9/82Jld.9(9)). Leopard 1 (8 year old) and Leopard 2 (11 year old) were the only captive individuals on the island of Borneo. They were housed in separate enclosures at Lok Kawi Wildlife Park, Kota Kinabalu, Sabah. Cages were roofed, allowing sunlight through the sides and situated away from human disturbance. The individual cages measured 6 × 6 × 4 m (length, width, and height, respectively), with cemented floor and a raised, wooden platform. Enrichment consisted of wooden structures for climbing and scratching. Starting in 2019, with the construction of a new and larger enclosure, the two leopards were rotated to utilize a single, large display area with vegetation and enrichment. They were fed once a day, a mixture of whole chicken and beef, supplemented with minerals and vitamins. Clean water was available ad libitum.

In terms of body condition, both adult males were healthy but one was younger and the older male has obvious pathology of his testicles. This was also confirmed by routine hemogram and serum chemistry. Their body weights were measured after each anesthesia, prior to electro-ejaculation. The average body weight (± SD) for Leopard 1 was 26.1 ± 3.4 kg and 25.4 ± 4.3 kg for Leopard 2 (see Supplementary File 1 for details).

### 2.2. Anesthesia

Animals were fasted 12–18 h before drug administration. Each male received an induction dose of 0.06 mg/kg medetomidine (medetomidine 40 mg/mL, Kyron Laboratories, South Africa) and 4 mg/kg ketamine-HCl (ilium ketamil 100mg/mL, Troy Laboratories, Australia) administered intramuscularly (IM) via blowpipe system (Telinject, GmbH, Germany). This resulted in a total volume of less than 1.5 mL of medetomidine/ketamine fitted in a 3 mL homemade syringe dart with a modified 18 G × 1½“ hypodermic needle with side port. Once the individual was approachable, body weight was recorded using a digital weighing scale. 

Animals were intubated with a size 8.0 I.D endotracheal tube guided by a Macintosh laryngoscope (Weichuang Medical Technology, China). Blood oxygen saturation (SpO2) was measured using a pulse oximeter (EDAN, USA) with the probe clipped on the tongue. End tidal CO_2_ (ETCO2) was measured using a mainstream capnometer (Masimo EMMA®, Spain). Body temperature was measured using a hand-held digital thermometer. Anesthetic depth was assessed by jaw tone, anal tone, palpebral reflex, pupillary light reflex, and respond to noxious stimulus (withdrawal reflex). Blood glucose level was measured using a portable hand-held glucose meter (Accu-Chek, Roche, Malaysia). An intravenous (IV) port was placed for anesthetic drug top-up and for fluid supplement at a rate of 10 mL/kg/hr. Following general anesthesia, leopards were immediately transferred to an adjacent room and placed on right lateral recumbency on an examination table. After observations, leopards were moved back to their cage and administered with atipamezole (alzane 5 mg/mL, Zoetis, Spain) intra muscularly at a dose five times the dose of medetomidine and allowed to recover undisturbed. Leopard 1 was anesthetized on ten occasions for reproductive examination and electro-ejaculations. Anesthesia was only performed seven times on Leopard 2 who sometimes appeared too stressed for the procedure (Table 1).

### 2.3. Examination of Reproductive Tracts 

Examination was carried out with the leopard on right lateral recumbency. Once the fur was clipped around the scrotum and prepuce, the site was thoroughly cleaned using normal saline and wiped dry. An examination of the testicles and penis was carried out for external lesions. Both testicles were palpated for consistency, mobility in the scrotum and symmetry. The testicular consistency score was based on firmness (distance the testicular tissue can be depressed) and resilience (springiness, characterized by the ability of the testicular tissue to return to normal shape after pressing) of the testes. The scores were graded into (1) very firm, (2) firm, (3) moderate, (4) soft, and (5) very soft [10]. 

Both testes were also measured for length (L; cranial–caudal), width (W; medial–lateral) and scrotal skin thickness, using a digital, sliding Vernier caliper (IP 54, China). Subsequently, the total testicular circumference was calculated. Each testicular volume was calculated after subtracting scrotal skin thickness, using a formula, L x W (2) x 0.524. The total testicular volume was determined by combining the volume of the left and right testes [11]. 

Transcutaneous ultrasonography was performed on both testicles using a Sonosite M-Turbo®, USA with an L52× linear probe (10–5 MHz transducer). The transverse and sagittal section of the testicles were scanned. The dimensions and any pathology were recorded. Subsequently, a transrectal ultrasound was performed to visualize and take measurements of the prostate.

### 2.4. Electro-Ejaculation 

Prior to the electro-ejaculation procedure, the rectum was emptied of fecal materials and well lubricated with non-spermicidal gel (K-Y Jelly, Johnson & Johnson Co., Arlington, TX, USA). The genital region was thoroughly cleaned with normal saline and wiped dry with paper towel or gauze. The prepuce was retracted with the thumb and index finger and the penis cleaned. Once the penis was secured inside a 50 mL conical graduated tube, the prostate gland was palpated to determine the depth of rectal probe insertion (~ 10–12 cm). The rectal probe was lubricated and gently pushed into the rectum and on the prostate glands. The dorso-ventral position of the electrodes was verified before any stimulation. The stimulations were done using an electro ejaculator with a standard rectal probe measuring 2.5 × 7 cm (diameter and length), consisting of three 4.6 × 56.8 mm electrodes (Seager®, Dalzell Medical Systems, USA). The stimulation consisted of two to three series of 1–5 volts. Each series was divided into 2–3 sets of 10 stimuli each, with each stimulation increasing gradually until the maximum of 5 volts [5]. Each stimulus lasted about 3 s, followed by 4–5 s of rest before the next stimulus. The quality of stimulations was also assessed based on the response of the hind legs’ contraction and penile erection. On two occasions, the stimulation was increased to 6 V. Occasionally, rectal massage was used at the start or in-between stimulation, to expel the semen.

### 2.5. Semen Evaluation

The semen evaluation followed the protocol described for carnivores [12]. Each fraction of semen ejaculated between the stimulation, was collected in a 20 mL glass vial and was examined subjectively, for appearance, volume, consistency, and pH. Semen was immediately transferred into a centrifuge tube and into a water-bath, at 37.5 °C. A volume of 3–5 µL of raw, undiluted semen was transferred onto a glass slide, covered with a cover-slip and examined under a phase contrast microscope (Olympus CX43-32P01, Japan) at a 200x magnification, for motility and mass activity (progressive motility percent). Sperm vigor was evaluated on a scale of 0–5. No motility was “0”; slight movement in >75% of sperm showing vibrations only was represented by “1”; moderate forward movement in >50% sperm was represented by “2”; progressively forward movement with ~70% sperm was represented by “3”. Sperm with 90% and >95% active motile sperm were represented by 4 and 5, respectively. Sperm motility was expressed as % of cells actively moving in a forward movement [13].

The forward progressive sperm motility (%) was measured by placing 10µl of semen on a prewarmed (37 °C) glass slide with a coverslip and 200 sperms were counted [13]. All fractions of ejaculate with sperm vigor of ≥2 were pooled together. Sperm concentration was determined in a Neubauer counting chamber after dilution of 2µL semen in 38 µL of formal saline (4% formaldehyde in saline solution), a dilution of 20x. Using the sperm motility (%) and the progressive motility, a sperm motility index (SMI) was calculated using the formulation previously described [12].

A ratio of one part semen to three parts Eosin-Nigrosin was adopted to avoid a too-high concentration of sperm on the thin smear, which makes it very difficult to view and count the live:dead sperms. A similar ratio of semen to Diff-Quik stain (USA) were processed for morphological examination. Morphologic defects observed in spermatozoa were classified as primary (disorders within the spermiogenic epithelium) or secondary (sperm after they have left the spermiogenic epithelium or due to semen mishandling). The primary sperm abnormalities included abnormal heads, nuclear vacuoles, structural abnormalities of midpiece or tail, coiling of midpiece, and cytoplasmic droplets. Secondary abnormalities included distal cytoplasmic droplets, detached heads, simple bent tails, and loosened or detached acrosomes [7,14]. Urine contamination was initially suspected based on the color (yellowish) and consistency (watery) of the semen. The pH strips (Whatman® pH indicator paper strip, USA) was used as an indicator of urine contamination, with reading of 6.0 being highly conclusive. The total semen volume was recorded at the end of the electro-ejaculation.

Semen fractions (sub-samples collected during different stimulations) of similar quality were pooled inside a 2 mL vial and mixed with Tris/Ham F-10 (comprising of fructose, citric acid, streptomycin and penicillin in one liter double distilled water; pH 7.5) in a ratio of 1 part of semen + 1 part of Tris/Ham F-10 and kept at room temperature in a dark environment. An aliquot of 2 µL semen mixture in 38 µL formal saline (4% formalin in saline) with a dilution factor of 20x was uploaded onto the hemocytometer and examined under 400x magnification for sperm concentration.

### 2.6. Cryopreservation 

Samples that exhibited a minimum motility of 20%, vigor of ≥2, and 30% normal sperm morphology were subsequently processed for cryopreservation. The semen was washed with Ham F-10 (Hepes + antibiotic)/Tris buffer and subjected to centrifugation at 300× *g* for 8–10 min. The supernatant was discarded and the sperm pellet was reconstituted to its initial volume with Tris and TYB (TEST-yolk buffer based extender with gentamicin sulphate and 12% glycerol; Irvine Scientific, USA) semen extender, in a ratio of 1:2 (i.e., 1 part of sperm pellet with Tris/semen extender-I, TYB 0% glycerol + 1-part semen extender-II, TYB 12% glycerol) giving a final glycerol concentration of 6% [7]. After cooling, sperm cells were evaluated for percent motility and sperm vigor, as previously described. Cooled samples that showed at least 30% motility and sperm vigor of ≥2 were then cryopreserved. Cooled semen was divided into aliquots of 13–29 × 10^6^ sperm/mL.

The total number of sperm per straw was adjusted to 10–20 × 10^6^ sperm/straw. Thereafter the aliquots were drawn into the 0.25 mL plastic straws, 100 µL/straw, and sealed with polyvinyl sealing powder. 

Semen straws were equilibrated from room temperature to 4 °C in a refrigerator for 2 h. A Styrofoam box (inside dimensions 33 × 24 × 23 cm was filled with liquid nitrogen to a depth of 4 cm. Subsequently, a 1 cm thick Styrofoam “boat” was floated on top of the liquid nitrogen for 10 min followed by placing the straws on top of the “boat” for another 10 min (cooling rate of ~220 °C/min) before plunging into liquid nitrogen [15,16]. The straws were then packed into goblet and stored in a liquid nitrogen tank. None of the samples from Leopard 2 were frozen because of the poor quality.

### 2.7. Thawing Procedure and Post-Thaw Analysis

Two frozen semen straws from Leopard 1 were thawed and evaluated for acrosomal integrity., Straws were removed from the liquid nitrogen using a long thumb forceps and held for 5–10 s in air before being immersed in a water bath (37.5 °C) for five minutes. The straws were wiped dry and emptied into a 2 mL vial (prewarmed at 37.5 °C) in a water bath and allowed to stand for 10 min before evaluation for general and progressive motility percentages. Acrosomal evaluation was carried out using a thin smear from a dilution consisting of 3 µL semen and 6 µL normal saline. The spermatozoa were examined under phase contrast microscope at 400x magnification. Note that acrosomal evaluations were not conducted on the fresh semen because of the absence of phase contrast microscope at the collection site. 

### 2.8. Statistical Analyses

Excel analysis (Toolpack package) was used to run data analysis including average, standard deviation, and Student *t* test. Normal distributions (Kolmogorov–Smirnov test) and homogeneity of variances (Bartlett’s test) were checked. When data were not normally distributed or variance was not homogeneous, Wilcoxon test were used to compare the values.

## 3. Results

### 3.1. Testicular Biometry 

Leopards presented testicles with variable consistency, symmetry, and mobility in the scrotum. Testicular consistency scores showed that Leopard 1 had firmer testicles in contrast to Leopard 2 which had more frequent scores of soft–flaccid (see Table 2 and Supplementary File 1 for details). The testicular circumference and volumes were higher in Leopard 2 than in Leopard 1. 

### 3.2. Ultrasonography of the Reproductive Tract

**Figure 1:**
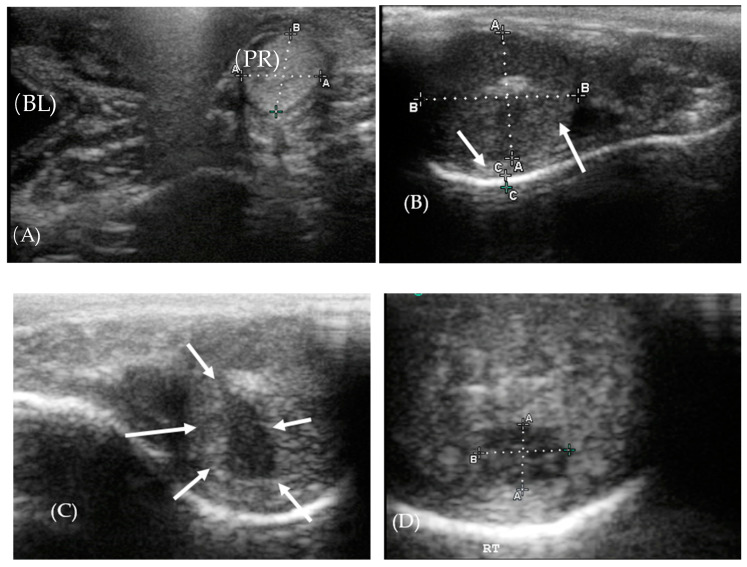
Ultrasonography of the testis and prostate. (**A**) The prostate gland (PR) was isoechoic to the bladder (BL). (**B**) Right testis of Leopard 1 showing the hydrocoele (solid arrow) and fibrosis (dashed arrow), beneath the tunica albuginea. (**C**,**D**) Hypoechoic area in the testis of Leopard 2 (solid arrows).

The oval-shaped prostates (Figure 1A), viewed in a transverse plane, averaged 1.2 ± 0.1 cm by 1.0 ± 0.1 cm for both leopards. Tissue had uniform echogenicity with a smooth and stippled texture. Testicles of both leopards did not have a uniform parenchymal echotexture and were more heterogenous in Leopard 2 as compared to Leopard 1 (Figure 1B–D). Both leopard testes had some degree of fibrosis or calcification as seen by the hyperechoic streaks beneath the tunica albuginea (Figure 1B,C). The mediastinum testis was visible as a hyperechoic central linear structure in the testis. A hydrocoele was visible near the tail of epididymis of the right testis of Leopard 1 (Figure 1B). The right testicle of Leopard 2 contained a hypoechogenic structure measuring 0.5 × 0.7 cm (Figure 1C,D). 

### 3.3. Semen Evaluation 

Sample pH for Leopard 1 was higher than for Leopard 2 (see Table 3 and Supplementary File 1 for details). Eight out of 10 ejaculates collected from Leopard 1 had a pH ≥ 8.0 with low pH around 6 on two occasions. This was better than for Leopard 2 that produced samples with pH < 7 during four collections out of seven (see Supplementary File 1 for details). On two occasions (one from each male), large amount of urine (5–7 mL) were collected at the end of the procedure. 

**Figure 2:**
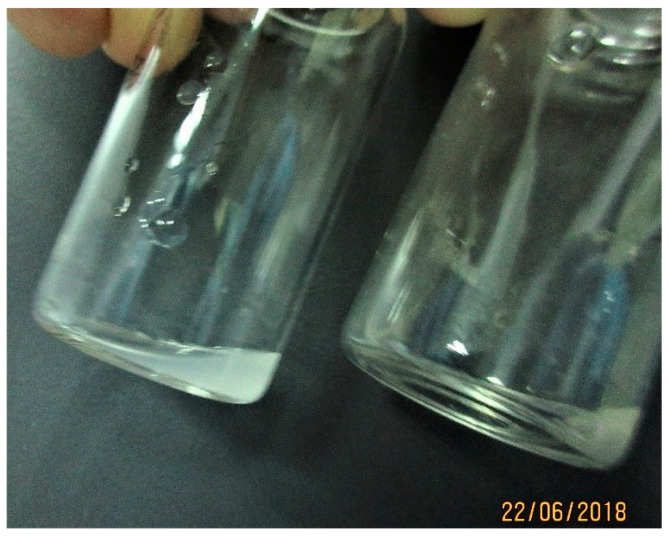
Semen collected from Leopard 1 in June 2018 showing the first (left) and subsequent collection (right).

Samples ranged in color from milky to translucent or clear. Samples from the first or second stimulation (rectal massage or 1–3 V electrical stimulation) was thick or concentrated and milky or white or whitish in color (see Supplementary File 1 for details). The subsequent ejaculates were thin, opaque or translucent (Figure 2). In Leopard 2 semen color was slightly whitish to translucent and sometimes yellowish (urine contamination), with a thin consistency. Leopard 1 presented a more milky, creamy or whitish semen, with a thicker consistency (see Supplementary File 1 for details).

Ten ejaculates obtained from Leopard 1 ranged from 250µL to 560µL, in contrast to leopard 2, 115–295 µL (Supplementary File 1). As an average, sample volumes were similar between Leopard 1 and Leopard 2 (Table 3). However, all samples from Leopard 1 were ≥250 µL, 90% exceeded 300 µL, 40% were >400 µL, and 20% were>500 µL. In contrast, 67% of samples from Leopard 2 were between 250 and 295 µL.

Sperm motility was better for Leopard 1 than Leopard 2 (Table 3). All samples collected from Leopard 2 were either azoospermic or containing dead or very low sperm concentrations and very low sperm motility index (Table 3). As a result, sperm concentration and total sperm output was much lower in Leopard 2 (Table 3). Percentage of sperm viability and morphologically normal cells were similar between Leopards 1 and 2 (Table 3). 

**Figure 3:**
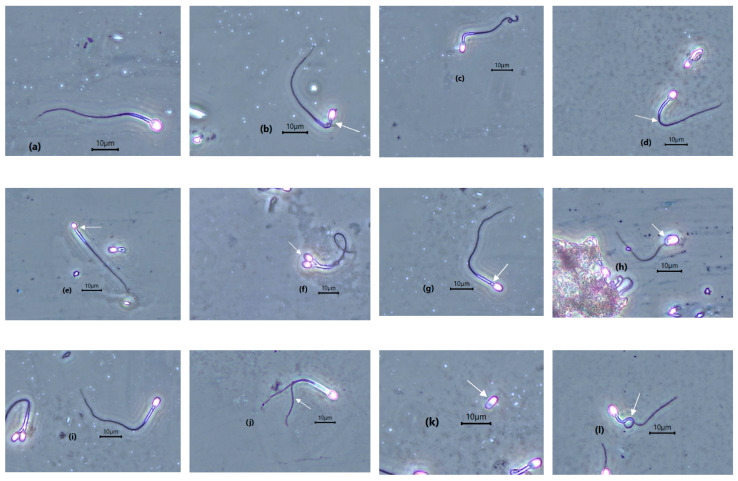
Photomicrograph image of Sunda clouded leopard spermatozoa under phase contrast microscopy (white arrows are pointing out the defects). (**a**) Normal spermatozoa, (**b**) bent midpiece, (**c**) coiled tail, (**d**) bent tail, (**e**) microcephalic, (**f**) bicephalic, (**g**) cytoplasmic droplet, (**h**) macrocephalic, (**i**) irregular shaped tail, (**j**) double tail, (**k**) detached head, and (**l**) shoe-hook tail.

Incidence of teratospermia in both leopards was high (Table 4). However, azoospermia and oligospermia were only observed in Leopard 2, and not in Leopard 1. Defective tails, comprising bent or tightly coiled tails, constituted the second-most-frequent structural abnormality in both leopards. Among these, tightly coiled tail was the most common, followed by bent tail and coiled tail. Midpiece defects represented the most frequent structural abnormality in both leopards. Sperm head defects, consisting of microcephalic, macrocephalic, and bicephalic were also observed. This morphological defect constituted the lowest incidence in the two Sunda clouded leopards (Table 4; Figure 3).

### 3.4. Post-Thaw Evaluation 

**Figure 4:**
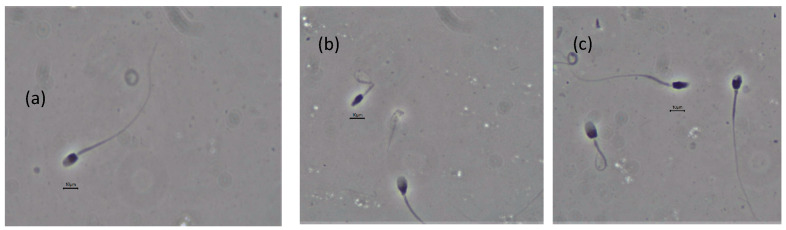

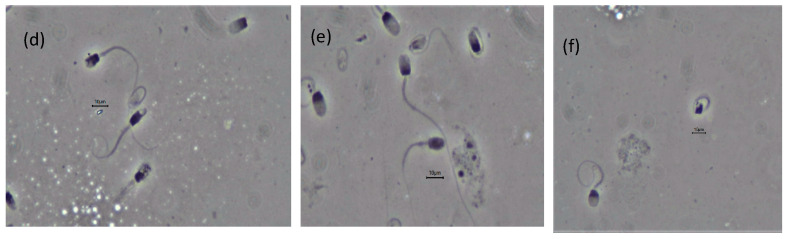
Normal acrosome (**a**), irregular distribution of acrosomal material (**b**,**c**), fragmented acrosome (**d**), loss of acrosome (**e**), and detached acrosome (**f**).

Both post-thawed semen samples from Leopard 1 showed a very low general and progressive motility (Table 5). The average abnormal acrosome comprised almost half of the spermatozoa evaluated (Table 5; Figure 4). 

## 4. Discussion

This is the first report about semen characteristics in the Sunda clouded leopard and the first attempt to freeze semen. The study showed that semen quality was constant in a healthy and young clouded leopard while semen quality was compromised in an older individual. The pathology within the testicles of the older leopard, does not impact its general health, but will significantly reduce the semen quality. Although post-thaw survival was low, enough motile sperm cells with intact acrosomes for potential in vitro fertilization can be recovered. 

Testicular consistency is a reasonable indicator of testicular function and semen quality. In general, the testicular consistency scores for the two Sunda clouded leopards, indicated Leopard 1 as having better firmness and tone of the testes when compared to Leopard 2. Although there are many factors that can contribute to the soft consistency in Leopard 2, a distinguishing factor between the two leopards is age. Leopard 2 being an older individual. Both leopards were managed under the same conditions. However, previous studies in buffaloes, guided with ultrasonography, showed that testicles with moderate consistency produced better semen quality as compared to firm or soft ones [17]. Similarly, in a study of reproductive disorders in domestic cats, soft testicle was associated with oligozoospermia and azoospermia [18]. In dogs, seminomas and interstitial cell tumors in the testicle are palpable as soft, cystic masses [19]. Some of the attributes of the hard testis could be fibrosis and crystal-like deposition in the testicular parenchyma as observed in other species [20]. In addition, animals with testicle consistency scores 2 and 3 generally produce good quality semen. Males with scores of 4 or 5 are likely to produce poor quality semen. Similarly, males with very firm/hard testicles may suffer fibrosis and may have unsatisfactory semen [21].

Leopard 2 had larger testes but semen quality was poor. The most likely reason for this is the pathology observed in the testis via ultrasonogram, suggestive of either neoplasia, degeneration, or testicular disease. A high prevalence of testicular diseases was observed in old dogs, and those diagnosed with severe degeneration were azoospermic and had impaired sperm release [22]. 

Although, both leopards showed some degree of heterogenicity of the testicular parenchyma, this echotexture was more pronounced in Leopard 2, which correlates to his poor semen quality. In humans, ultrasonographic heterogeneity of the testis, was shown to corresponds histologically to regions of grade 2 or 3 tubular atrophy and sclerosis. The incidence is encountered in middle aged and elderly, above 60 years of age, leading to partial or complete loss of spermatogenesis [23,24]. However, in a recent study with dogs, no association was found between testicular heterogenicity and semen quality [25]. In addition, leopard 2 had a well-defined, hypoechoic mass beneath the tunica albuginea of his right testis, indicating an underlying pathology which disrupts spermatogenesis [26,27]. This growth, mass, or fluid would alter the size of the testis, making it larger instead of smaller as in cases of atrophy due to age. In dogs, incidence of testicular interstitial fibrosis and germ cell degeneration/depletion were pronounced in those over 9 years of age [28]. The causes of testicular atrophy/degeneration in domestic cat are heat from fever, drugs, poor health and debility, hypervitaminosis A, and advancing age [29,30]. 

The genera *Neofelis*, clouded leopard and *Panthera*, lion have the highest percentage of morphologically-abnormal spermatozoa [12]. The present evaluation will contribute to the establishment of baseline data for sperm parameters in Sunda clouded leopards. The study demonstrated similar findings of teratospermia, with mainland clouded leopards [9,12,20]. In addition, the low semen quality was more prominent in the older leopard (leopard 2), with smaller volume of ejaculates and lower semen pH, motility, concentration, and viability. A decline in reproductive function is a well-known feature of aging in mammalian species [11,31]. Several studies revealed an age-dependent decrease in free testosterone concentrations in the human male with unchanged or increased estradiol concentrations and increased concentrations of luteinizing and follicular stimulating hormones [23,32]. Similarly, old dogs exhibited lowered epididymal sperm motility, sperm vigor, and viability [28]. 

A high incidence of pleiomorphic spermatozoa in clouded leopards were observed in previous and recent studies, suggesting the influences of photoperiods, husbandry, nutrition, housing, and breeding protocols [5,20]. In the study, there was no significant difference in semen quality between the leopards, throughout the year. In the present study, the correlation between months and semen volume is not significant (*p* = 0.05). However, without an estrus female, this factor of breeding season cannot be verified at the zoo. Nevertheless, a basic parameter that can attribute to good semen quality is the presence of positive stimulation from a sexually-active female. It was demonstrated that, paired male clouded leopard have a tendency to produce better semen quality as compared to those housed singly [5]. In the present study, both the male leopards were never paired (housed singly) and housed next to each other. There was never a female at the zoo. In most male mammalian species, the normal reproductive function is dependent on a dynamic and integrative hypothalamic–pituitary–testicular axis (HPT). Positive extrinsic stimuli, including sight, smell, and physical contact with an estrus female will stimulate the HPT axis, starting a series of physiological responses, targeting the testis, causing increase production of testosterone and androgen [6,33]. In the wild, recent studies showed that scent-marking behaviors are an integral part of intraspecific communication and social interaction in the wild Sunda clouded leopard. Males overlapped in their home range, competing at scent marking areas, reinforcing territories and attracting mates [34].

Nutrition plays an important role in animal health and reproduction. This was clearly demonstrated in an international conservation project between zoos in the United States and Thailand. The dietary improvements in four species of wild felids in zoological institutions in Thailand over more than a year, showed a marked improvement in sperm production and quality [35].

Frozen-thawed sperm samples from Leopard 1 had low motility but incidence of normal acrosome was acceptable and compatible for future in vitro fertilization. Poor semen quality, after thawing confirmed the high cryosensitivity characteristic of spermatozoa in the clouded leopards as in other species of Felidae, suggesting species specificity [5,36]. In addition, the high incidence of teratospermia in fresh ejaculates of most non-domestic felids resulted in poor quality post-thaw semen. The etiology for teratospermia is unknown but is associated with extreme genetic monomorphism, diminished genetic variation, and low gene diversity. Despite the above reasons, the individual effect may also impact cryo-sensitivity in the clouded leopards (34). Recent studies comparing simple washing and single-layer centrifugation of post-thawed clouded leopard semen also demonstrated a low percentage of intact acrosomes [5]. 

## 5. Conclusions

Although based on two individual Sunda clouded leopards, the present study demonstrated similarities between mainland clouded leopards with regards to structurally-abnormal morphology of fresh and frozen-thawed semen. Sperm selection protocol using colloid centrifugation should be utilized in future semen collection from the Sunda clouded leopard. Data in this first study will address the urgent need to improve breeding management and increase captive population through conservation breeding and assisted reproductive technology. The protocols for semen collection and cryopreservation also need to be optimized in the future. Lastly, the very small number of captive Sunda clouded leopards within the ranged states strongly justifies a collaborative program like the one initiated between Zoological Park Organization in Thailand and zoos in the United States. 

## Figures and Tables

**Table 1:** Dates of anesthesia and electro-ejaculation procedures in two Sunda clouded leopards living singly.

Date	Leopard 1	Leopard 2
15 January 2018	Anesthesia and electro-ejaculation	Anesthesia and electro-ejaculation
28 February 2018	Anesthesia and electro-ejaculation	Anesthesia and electro-ejaculation
18 April 2018	Anesthesia and electro-ejaculation	Anesthesia and electro-ejaculation
22 May 2018	Anesthesia and electro-ejaculation	Anesthesia and electro-ejaculation
22 June 2018	Anesthesia and electro-ejaculation	No collection
9 August 2018	anesthesia and electro-ejaculation	Anesthesia and electro-ejaculation
20 September 2018	anesthesia and electro-ejaculation	No collection
9 October 2018	anesthesia and electro-ejaculation	Anesthesia and electro-ejaculation
20 November 2018	anesthesia and electro-ejaculation	Anesthesia and electro-ejaculation
3 December 2019	anesthesia and electro-ejaculation	No collection

**Table 2:** Mean (± standard deviation) of length, width, and volume of right–left testes and total testicular volume of two Sunda clouded leopards living singly.

Leopard	Testis	Testicular Consistency Score	Length (cm)	Width (cm)	Circumference of Testis (cm)	Volume (cm^3^)	Total Volume (cm^3^)
1	Right	1.8 ± 0.7	3.3 ± 0.3	2.3 ± 0.2	7.2 ± 0.6 ^a^	9.5 ± 1.4 ^a^	18.9 ± 1.7 ^a^
	Left	1.8 ± 0.7	3.5 ± 0.4	2.3 ± 0.2	7.2 ± 0.6 ^a^	9.5 ± 1.0 ^a^	
2	Right	3.0 ± 1.7	3.6 ± 1.0	2.7 ± 0.4	8.7 ± 1.1 ^b^	13.8 ± 2.7 ^b^	24.8 ± 4.6 ^b^
	Left	2.8 ± 1.5	3.4 ± 0.6	2.5 ± 0.3	7.5 ± 0.4 ^ab^	11.0 ± 3.3 ^ab^	

In each column, values with different superscripts (a, b) were statistically different (t-test; *p* < 0.05).

**Table 3:** Semen characteristics (mean ± SD) of two Sunda clouded leopards living singly.

Semen Characteristic	Leopard 1 (10 Samples)	Range (10 Samples)	Leopard 2 (3 Samples)	Range (3 Samples)
pH	7.9 ± 0.9 ^a^	5.5–9.0	6.8 ± 1.1 ^b^*	5.0–9.0
Volume (µl)	402 ± 92	230–560	326 ± 197 **	115–700
Motility (%)	54.5 ± 24.2 ^a^	10–85	2.4 ± 3.5 ^b^	0–10
Progressive motility (%)	43.2 ± 23.5 ^a^	7–80	1.0 ± 1.7 ^b^	1–5
Sperm motility index	54.3 ± 24.9 ^a^	22.5–82.5	12.8 ± 1.65 ^b^	11–15
Sperm concentration (×10^6^/mL)	122.4 ± 84.7 ^a^	51.3–304.4	6.8 ± 3.38 ^b^	3.3–11.2
Total sperm (×10^6^)	44.6 ± 24.8 ^a^	13.7–97.4	1.8 ± 0.42 ^b^	1.3–2.3
Viability (%)	55.2 ± 18.2	28.7–73.9	52.1 ± 14.1	35.6–70
Normal morphology (%)	23.9 ± 12.3	8.7–47.6	31.2 ± 9.1	23.3–44

In each row, values with different superscripts (a, b) were statistically different (t-test; *p* < 0.05). * Value calculated on six samples; ** Value calculated on five samples.

**Table 4:** Morphology of abnormal spermatozoa (average ± SD) of two Sunda clouded leopards living singly.

Morphology (%)	Leopard 1 (10 Samples)	Range	Leopard 2 (3 Samples)	Range
Abnormal sperm	75.8 ± 12.0	52.4–91.3	68.8 ± 9.1	56–76.7
Macrocephalic	0.6 ± 0.9	0.0–3.0	0.3 ± 0.2	0.0–0.5
Microcephalic	2.6 ± 4.1	0.0–14	4.0 ± 1.7	1.7–5.4
Bicephalic	0.17 ± 0.3	0.0–0.9	0.3 ± 0.2	0.0–0.5
Mid piece defect	27.9 ± 7.3	15.8–39.13	28.7 ± 3.5	24–32.3
Bent tail	15.1 ± 7.6	4.4–32.6	16.4 ± 4.7	10–21.3
Coiled tail	6.2 ± 10.8	0.0–38.3	6.8 ± 3.4	1.3–8.5
Tightly coiled tail	21.8 ± 14.9	0.0–45	14.9 ± 3.2	11–18.8

**Table 5:** Sperm characteristics in two different frozen-thawed semen from Leopard 1. Sample 1 was collected on 15 January 2018 and Sample 2 was collected on 28 February 2018. Both were stored for more than 2 years before thawing and evaluation.

Semen Characteristics (%)	Sample 1	Sample 2
General motility	10	5
Progressive motility	3	5
Sperm viability	13.6	11.6
Normal spermatozoa	21.5	40.2
Normal acrosome	42.7	65.8

## Supplementary Materials

The following are available online at https://www.mdpi.com/2076-2615/10/6/1072/s1, Table S1: Semen characteristics of two Sunda clouded leopard at Lok Kawi Wildlife Park, Sabah.
